# High Mobility and STIs/HIV among Women Informal Cross Border Traders in Southern Mozambique: Exploring Knowledge, Risk Perception, and Sexual Behaviors

**DOI:** 10.3390/ijerph17134724

**Published:** 2020-06-30

**Authors:** Joana G Salia, Mohsin Sidat, Sónia F Dias, Maria R O Martins, Isabel Craveiro

**Affiliations:** 1 Instituto Superior de Ciências de Saúde—ISCISA, 0101 Maputo, Mozambique; 2 International Public Health and Biostatistics Unit, Institute of Hygiene and Tropical Medicine, NOVA, University of Lisbon, 1349-008 Lisbon, Portugal; mmsidat@gmail.com (M.S.); mrfom@ihmt.unl.pt (M.R.O.M.); isabelc@ihmt.unl.pt (I.C.); 3 Global Health and Tropical Medicine, GHTM, Instituto de Higiene e Medicina Tropical, IHMT, Universidade NOVA de Lisboa, UNL, 1349-008 Lisboa, Portugal; 4 Faculty of Medicine, Eduardo Mondlane University, 0101 Maputo, Mozambique; 5 NOVA National School of Public Health, Public Health Research Centre, Universidade NOVA de Lisboa & Comprehensive Health Research Center (CHRC), 1600-560 Lisboa, Portugal; smfdias@yahoo.com

**Keywords:** women, informal, cross-border, traders, circular mobility, behaviors, knowledge, STIs/HIV

## Abstract

High circular mobility creates vulnerability and elevates risk for sexually transmitted infections (STIs) including Human Immunodeficiency Virus (HIV). We aim to explore knowledge, perceptions of risk, and sexual behaviors in relation to STIs/HIV, in Mozambican women involved in an informal cross border trade (ICBT) and residing in South Mozambique. A cross-sectional quantitative study, in 200 women cross border traders (WICBT), affiliated to the *Mukhero* Association, using a structured, face-to-face questionnaire, was conducted. Descriptive statistics and Pearson’s Chi-square test were used. The median age of participants was 37.0 years (interquartile range (IQR): 31.0–43.0), 100% were literate, travelled on average six times a month. WICBT with a high education level were more likely to have awareness of Gonorrhea, Syphilis, and Candidiasis; to self-perceive being at risk of getting HIV, Syphilis, and Human Papilloma Virus (HPV); and to test for HIV and Syphilis. Those with a low education level were more likely to have misconceptions about HIV and ever have sex in exchange for money/goods/services. Married participants were more likely to know how to prevent HIV. Participants with a high income were more likely to know about HPV; to self-perceive being at risk of getting Syphilis; to point sex workers as being at higher risk of getting HPV; and to ever test for HIV. WICBT with a low income were more likely to have sex in exchange for money/goods/services. Low and inconsistent knowledge and misconceptions of STIs/HIV, high sexual risky behavior, low perception of risk of getting STIs/HIV among this neglected and key population suggests their increased vulnerability to the STIs/HIV.

## 1. Introduction

The involvement of women in Informal Cross-Border Trading (ICBT) in the context of high mobility and circular migration has become an increasing phenomenon in the sub-Saharan Africa, especially the Western, Eastern, and Southern Africa (SADC) sub-regions [1,2].

The ICBT practiced by 70–80 percent of women, contributes towards supporting families, reducing poverty and unemployment, empowering women, and growth of their countries’ economies [3,4,5]. Despite this contribution, women in informal cross-border trading (WICBT) suffer from stigmatization, poor transportation, corruption, extortion, robbery, confiscation of goods, long queues and delays at the borders, various forms of gender-based violence, and sexual harassment by customs officials and truck/bus drivers [3,4,5,6,7].

Evidence shows that these women devise coping strategies for economic survival during their migration process, such as paying bribes to the customs officers, cooperation among women, and engaging in high sexual risky behavior (high number of sexual partners and frequency of multiple concurrent partners, transactional sex) [8]. Some of these coping strategies create risk and vulnerability for sexually transmitted infections (STIs) including Human Immunodeficiency Virus (HIV) [9,10,11].

In Mozambique, ICBT involves mostly women (71%) [12], and this activity is known locally as *Mukhero*. The ICBT is done in a circular way (a constant round-trip movement) to neighboring countries that are heavily affected by STIs/HIV. This poses major challenges to sexual and reproductive health because of the practices and behaviors used by some women [7,13].

Migrants and high-mobility people have been referred to facilitate the spread of tuberculosis, malaria, and sexually transmitted infections including human immunodeficiency virus, especially women. The impact of sociocultural patterns of mobility on health includes factors such as: Gender, age, ethnicity, cultural norms and sociocultural and economic status, lower availability and reduced access to health services [9,14,15]. 

The STIs, which was the focus of this research, remain a major public health problem worldwide, but in particular in developing countries, such as Mozambique. Women are more likely to suffer the consequences of STIs including pelvic inflammatory disease, cervical cancer, infertility, ectopic pregnancy, and they have an impact through their role in facilitating sexual transmission of HIV [16]. 

Studies in Eswatini, a neighbor country of Mozambique, where the *Mukheristas* cyclically travel, have shown that the estimated prevalence of chlamydia, gonorrhea, trichomoniasis, Syphilis, and HIV among migrant’s women of reproductive age was 7%, 6%, 1.4%, 18.8%, and 42.7%, respectively. Additionally, the risk factors associated with those STIs/HIV were Human Papilloma Virus (HPV) positive, not using condoms, and being self-employed [17].

In Mozambique, a recent study showed that the prevalence of *Neisseria gonorrhea* (NG), *Chlamydia trachomatis* (CT), Syphilis, and HPV was 14%, 8%, 12%, and 12%, respectively [18]. Another study in the Mavalane area of Maputo city and at the Manhiça Health Centre District, showed that 2% of women tested positive for NG, 4% for CT, 8% for *Trichomonas vaginalis* (TV), 17% for HIV, and 8% of active Syphilis in the Manhiça district [19]. Furthermore, results from the major hospital of the country, in Maputo city, demonstrated that the prevalence of STIs among women, were: *C. trachomatis* (6.5%), *bacterial vaginosis* (34%), *T. vaginalis* (2%) Syphilis (5%), and HIV (35%) [20].

Female circular mobility may contribute to the sustained high prevalence of STIs and HIV in Southern and East Africa [14], because mobility favors a high risk sexual behavior and creates opportunities for sexual networking [14,21].

In the Southern Africa region, a higher prevalence of HIV/AIDS (AIDS—acquired immunodeficiency syndrome) was found in the main high mobility corridors [22], including the Maputo–Witbank corridor, where the participants travel through. A study conducted by the IOM in South Africa in 2010, showed that the HIV prevalence in agricultural workers from Eswatini was 52.3%, 51.3% in South Africa, and 43.2% in Mozambique, highlighting the great importance of so-called “Spaces of vulnerability”, ranging from the place of departure, transit, and destination. The knowledge of these spaces led to assume that people are subject to: (1) Long stay away from home varying from days to months; (2) delays and long stay at borders, where high risk behaviors are common, with duration from a few hours to days; (3) improvised accommodation along borders and agricultural fields; (4) limited access to information and health services [23]. 

In Mozambique, two national population-based surveys, INSIDA (2009) (INSIDA: Inquérito Nacional de SIDA (AIDS National Survey)—first population based survey carried out in 2009) [24] and IMASIDA (IMASIDA: Inquérito de Indicadores de Imunização, Malária e SIDA (Nacional Survey for Indicators for Imunization, Malaria and AIDS)—population based survey carried out in 2015) (2015) [25], showed an overall prevalence of 11.5% and 13.2% among men and women aged 15–49 years, respectively. The disproportionate impact of the HIV epidemic in women has been attributed to: Poverty; multiple sexual partners; transactional sex; inconsistent or no use of condoms; gender inequality; violence against women; cultural practices; and migration [22]. For example, the results of the study held in the Chókwè district, Southern Mozambique, showed that the prevalence of HIV was 29.4%, and it was associated with migrant labor patterns, placing all active women at a higher risk for HIV infection [26]. 

The levels of knowledge about HIV were high among men and women, in the general population aged 15–49 years in Mozambique. However, there was a low percentage (32%) of adults with comprehensive knowledge about the HIV/AIDS [24]. Moreover, a study conducted by the IOM (2010) [27] in the SADC region involving South African and Mozambican migrant workers in the Limpopo and Mpumalanga provinces, showed that there was low knowledge and low perception of risk and vulnerability about HIV/AIDS, high prevalence of risk behaviors, inconsistent condom use, and increased vulnerability of women. 

The debate about the need to intensify the promotion of the simultaneous use of contraceptive methods including the condom is increasing, with a view to confer dual protection against STIs and unwanted pregnancies in women with a high risk to STIs/HIV [28]. 

Several studies were carried out in Mozambique on economics, socio-anthropologic, and on health issues concerning the transmission of STIs/HIV among migrant’s miners, truck-drivers, and sex workers. However, there is scarcity of published studies on women involved in ICBT. In particular, knowledge is lacking on context and practices that put these women involved in ICBT at higher risk of STIs/HIV and unwanted pregnancies. Thus, with this study, we aimed to explore knowledge, perceptions of risk, and sexual behaviors in relation to STIs/HIV, in Mozambican women involved in an informal cross border trade (ICBT) and residing in Southern Mozambique. 

## 2. Material and Methods

### 2.1. Study Design and Participants

This was a cross-sectional quantitative study conducted from February to September 2015. The study involved 200 cross-border informal trade women (*Mukheristas*) aged 18 to 49, members of the *Mukhero* Association, who cross the borders of Ressano Garcia, (border post with South Africa), Namaacha and Goba (both bordering Eswatini), living in Maputo City (country capital) and Maputo Province, southern Mozambique. 

**Figure 1:**
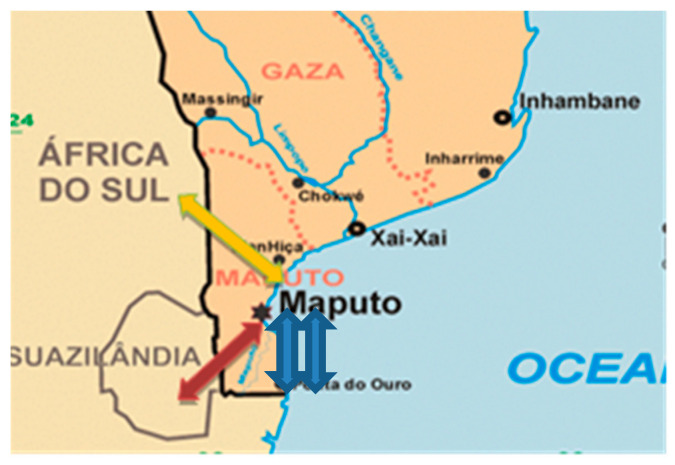
Map of southern Mozambique with the borders of Ressano Garcia, Namaacha, Goba, and Ponta de Ouro.

Maputo City is the capital of Mozambique, with 1,120,867 inhabitants, corresponding to 4% of the general population, of which 52% are women. Maputo Province, which is a satellite of Maputo city, is inhabited by about 1,968,906 people (7.1%) [29]. It is in this province that the four borders are located, Ressano Garcia, Namaacha, Goba, and Ponta de Ouro, through which *Mukheristas* women cross to neighboring countries such as Eswatini and South Africa (see Figure 1).

### 2.2. Data Collection

Data collection took place at the *Mukhero* Association. Initially, a pilot study was conducted involving 10 women *Mukheristas* reselling their wares in the Fajardo market in Maputo City. This study was done to test our research instrument for further refinement. A systematic random sampling was used, based on the records of the *Mukhero* Association database. The women were interviewed at the *Mukhero* Association headquarters, in the markets where they sell and resell their imported products, freight unloading terminals, warehouses, in their homes, car parks, through face-to-face and a structured questionnaire in Portuguese language, by a previously trained team. 

### 2.3. Measures 

The question consisted of sociodemographic characteristics and *Mukhero* activities that included age, marital status, education level, household size, main occupation, income from *Mukhero,* motivations for practicing *Mukhero*, start of the practice of *Mukhero,* and number of monthly trips. Other questions evaluated the knowledge of STI/HIV such as if they have heard about HIV, Syphilis, HPV, Gonorrhea, Trichomoniasis and Candidiasis (Yes/No); ways of transmission of STIs, and ways of prevention; sexual risk behavior (Yes/No); multiple sexual partners, ever had sex in exchange for money/goods/services, and no use of condom (Yes/No); risk perception of getting STI/HIV (High risk/Low risk); reported lifetime STI’s testing (Yes/No); and signs and symptoms (Yes/No).

### 2.4. Data Analysis

Data were analyzed with SPSS (Statistical Package for Social Sciences) version 23 (IBM Corp., Armonk, New York, NY, USA). Descriptive statistics looking at the distribution of the variables such as frequencies, means, and median (interquartile range = IQR), were performed. Then, Chi-square (χ^2^) tests were calculated to understand the significance of the relationships found between variables. Differences were considered significant at a *p*-value of 0.05 or less.

### 2.5. Ethical Considerations

The research protocol was submitted and approved by the Ethics Committees of both the Institute of Hygiene and Tropical Medicine in Portugal and the National Bioethics Committee for Health in Mozambique (Ref. 169/CNBS/2014). The research was conducted on the basis of informed consent of the participants. After the data were collected, they were anonymized to ensure their confidentiality. In this way, we guarantee that there were no risks for the participants in the study.

## 3. Results

### 3.1. Description of the Study Participants and Mukhero Activity

Table 1 shows the sociodemographic characteristics, motivation for the practice of *Mukhero* and trips’ frequency. This study showed that for a total of 200 women enrolled in the study, the majority had an average age of 37 (IQR = 31.0–43.8) years, and they are married or live in common law marriage (59.5%), all can read and write. The main motivations for *Mukhero*’s practice were: Lack of schooling (13.5%), unemployment (24.5%), increasing in family income (23%), and low partner wage (14.5%). Most of the participants started the activity of *Mukhero* between 5–10 years and make between three and six trips per month. The main occupation (80.0%) of most of the participants is the *Mukhero* activity and they make from their business the equivalent of up to three minimum wages for their survival. Note that the minimum wage at that time in Mozambique was 4.851.84 Meticais, where 1 USD was equal to 44.37 Meticais.

### 3.2. Knowledge about Sexually Transmitted Infections including HIV/AIDS

Table 2 shows that all participants have heard about HIV/AIDS and 87% have heard about gonorrhea. While 56% and 41% have heard about cervical cancer and syphilis, respectively, and only a minority (19%) have heard about candidiasis and trichomoniasis (15%). 

According to Table 3, 94% of respondents know that HIV can be sexually transmitted, have unprotected sexual intercourse (96%), and using of contaminated needles (91.5%). However, we have to point out that although a majority (63%) of the participants stated that mosquito bites are not the common form of HIV transmission, 22% of participants think it is possible. On the other hand, only 17% of *Mukheristas* know how syphilis is transmitted and more than ninety percent of our interviewees do not know how it is transmitted or how one can be prevented from being infected by HPV. In relation to testing, the majority (85%) of the participants underwent HIV testing. However, it turns out that only 10% of respondents reported having tested syphilis and HPV, although syphilis screening was offered to all pregnant women in Mozambique.

As for prevention, more than 90% of participants know that condoms and having a single uninfected sex partner prevent HIV transmission. However, it should be noted that 19% of the *Mukheristas* reported that having sex with virgin girls and boys was one of the forms of prevention. On the other hand, only 18% and 8% of participants know how to prevent syphilis and HPV, respectively.

### 3.3. Perception of Risk to STIs

Regarding the level of self-perception about the possibility of contracting HIV within the *Mukheristas*, 66.5% of the interviewees consider that there is a lot of possibility, 27.5%, little possibility, and 6% did not answer. Considering also the possibility of contracting syphilis and HPV among the *Mukheristas*, 82.5% and 86.3% respectively did not know how to answer the question, as illustrated by Table 4.

### 3.4. Sexual Risk Behaviors, Reported Signs, and Symptoms of STIs 

Regarding the number of sexual partners per woman in the 30 days prior to the survey, Table 5 shows that 78% of our respondents had one sexual partner, 11% had two partners or more. Fifteen percent (15%) had occasional sex, of these, only 53.3% used the condom.

Analyzing the question “if they ever had sex in exchange for money, goods or services?”, 7.5% said yes. Of these, 99.5% was in exchange for services (facilitation of goods crossing at the border level, cargo loading, and long truck drivers). Moreover, in the 30 days prior to the survey, 19% of the participants reported pain in the lower abdomen, 17% vaginal discharge, 9% genital pruritus (itching), and 3% ulcer/wound/genital tumor.

The bivariate relationships between level of education, marital status, income from *Mukhero*, and knowledge, risk perception, lifetime STI’s testing, risky sexual behaviors about STIs/HIV, using Pearson chi-square independent tests, are shown in Table 6. We only presented the results that are statically significant.

### 3.5. Knowledge, Risk Perception, Lifetime STI’s Testing, Risky Sexual Behaviors about STIs/HIV, and Level of Education 

Participants with a high level of education were significantly more likely to have heard about Gonorrhea (96% vs. 84.6%, *p* < 0.001), Syphilis (66% vs. 34%, *p* < 0.001), Candidiasis (34.7% vs. 14.79%, *p* < 0.001), and know that having sexual intercourse with unprotected multiple partners spreads Syphilis (91.7% vs. 78.3%, *p* = 0.006). Contrarily, low literate participants were more likely to have had misconceptions such as a mosquito’s bite spreads HIV (31.75% vs. 8.9%, *p* < 0.001), having sex with virgin young boys and girls prevents against HIV (25% vs. 2.2%, *p* = 0.038; 28.6% vs. 2.2%, *p* = 0.007), respectively. Moreover, literate participants were more likely to self-perceive about being at risk of getting HIV (86% vs. 61.2%, *p* = 0.015), Syphilis (24% vs. 10%, *p* < 0.001), and HPV (17.1% vs. 1.1%, *p* = 0.010) than those with a low level of education. Additionally, participants with a high level of education were significantly more likely to lifetime test for HIV (94% vs. 82.6%, *p* = 0.006), and Syphilis (22.7% vs. 7.5%, *p* = 0.003), as compared to participants with a low level of education. Ever had sex in exchange for money/goods/services (11.1% vs. 0%, *p* = 0.010) by *Mukheristas,* were significantly associated with a low education level. 

### 3.6. Knowledge about STIs/HIV and Marital Status 

Participants that were not currently married were more likely to hear about Trichomoniasis (21.8% vs. 11.1%, *p* = 0.028) and Candidiasis (24.4% vs. 11.1%, *p* = 0.021), and were more likely to know how to prevent HIV (97.5% vs. 86.6%, *p* = 0.047). Meantime, participants that were currently married showed misconceptions such as having sexual intercourse with virgin young boys prevents HIV (26.5% vs. 8.3%, *p* = 0.038).

### 3.7. Knowledge, Risk Perception, Lifetime STI’s Testing, Sexual Behaviors about STIs/HIV, and Income from Mukhero 

Finally, women *Mukheristas* with a high income from *Mukhero* were also more likely to have heard about HPV (57.1% vs. 56.4%, *p* < 0.001), to self-perceive to be at high risk of getting HPV (76.6% vs. 76.2%, *p* = 0.041) and Syphilis ((31.1% vs. 10.5%, *p* = 0.022), to point sex workers to be at high risk of getting HPV (100% vs. 93.8%, *p* = 0.036), and to ever test for HIV (93.1% vs. 84.1%, *p* = 0.011) than those with low income from *Mukhero*. Contrarily, participants who had a low income were more likely to know that having sex with one uninfected partner prevents Syphilis (95.2% vs. 79.3%, *p* = 0.027) and to have sex in exchange for money/goods/services (8.8% vs. 0%, *p* = 0.042).

## 4. Discussion

The mobility of women informal cross border traders in Mozambique is locally well-known but not yet documented in the literature from a socio-epidemiological point of view. While the behavior, STIs/HIV risk factors of mobile men (trucks drivers, miners) as well the sexual workers, knowledge, sexual behaviors, perceptions of risk and vulnerability, reported signs and symptoms, and reported testing in relation to STIs/HIV, in Mozambican women involved in an informal cross border trade in Southern Mozambique, remain largely unknown.

Our study on high mobility and circular migration showed that these women made an average of six trips per month which is consistent with those seen among highly mobile populations in other researches. Surprisingly, we did not observe any significant relationship between numbers of trips with knowledge, sexual behaviors, perceptions of risk and vulnerability, reported signs and symptoms, and reported testing in relation to STIs/HIV. However, frequency of travel may repeatedly expose these women to the risk of contracting sexually transmitted infections including HIV, gender-based violence, and sexual harassment [3,5,6,8].

Contrary to what has been stereotyped in relation to these women with high mobility and circular migration, according to our findings the majority of them are married or in common law marriage, and almost had universal complete secondary school, although they referred to the lack of schooling, as motivation for the practice of *Mukhero.* Similar profiles were founded in the studies in Lesotho, Nigeria, and Portugal [9,30,31], where women who travelled frequently and made five or more trips were more likely to be married and have a higher level of education, compared to non-migrant. In our context, on the one hand, it may have been due to the massification of literacy campaigns among women made after the country’s independence, in 1975. On the other hand, it might be explained by the nature of their activity. These characteristics could result in high levels of knowledge that would assist them in the adoption of safe sexual behavior and a greater perception of risk and vulnerability to sexually transmitted infections during the process of high mobility. 

Our results revealed that HIV was the most commonly heard infection among STIs, followed by Gonorrhea and HPV. However, knowledge about the ways of transmission and prevention of HIV was inconsistent. Women with a low level of education presented more misconceptions and myths towards HIV transmission and prevention than those with a high education level. Misconceptions such as the mosquito’s bite spreads HIV, having sex with virgin young boys and girls prevent HIV were higher among low literate participants. Similar evidence has been found in other studies [32,33,34].

Despite considerable numbers (54.5%) of participants had already heard about the HPV, the majority of participants were unaware of the forms of transmission and prevention, the causative agent of the disease, and they did not perform screening. In line with our findings, a recent study [35] held in Mozambique, where knowledge about cervical cancer (CC) and vaccine acceptability in young adolescents aged 10–19 years has been evaluated, found that most of the participants had already heard about CC, only one third knew the causative agent. 

HIV-HPV co-infection in sub-Saharan Africa, where STIs/HIV prevalence is high, may exacerbate the dynamics of control of the HIV epidemic in the subcontinent, especially in the SADC region [17,21,23]. This situation may delay the ambitious UNAIDS “90-90-90” targets that aim to end the HIV/AIDS epidemic by 2030, that is, 90% of all HIV-positive persons will know their HIV status, 90% of those with a diagnosis of HIV infection will receive sustained ART, and 90% of persons receiving ART will have viral suppression [36].

Although syphilis testing is a routine in the consultations of pregnant women seeking public pre-natal care in Mozambique, less than half of our study participants have heard about it, and only a minority reported having screened the agent that causes syphilis. This contrast may indicate that women attending these services have probably not been adequately explained about these procedures, emphasizing the importance of good communication in the care of these women. Study [37] in another context, but with Africans migrants, showed that communication difficulties between health professionals and migrant patients due to language differences and cultural practices of migrant women, were evident in stereotyped attitudes, discriminatory aspects, and lack of preparation of some health professionals.

The self-perceived risk to acquiring HIV is relatively high in participants with a high educational level. A previous published study in the Asian context [32], showed that respondents who had high knowledge on STIs/HIV might have felt more at risk because they knew that they might get infected, and they might apply their knowledge to reduce transmission, increase prevention, and behavior exchange. 

On the other hand, the majority of the *Mukheristas* participating in this study demonstrated a low level of self-perception in relation to other STIs. When we addressed “who could be in danger of contracting syphilis, gonorrhea, infection by HPV and trichomoniasis” among the following groups: Female sex workers, long truck-drivers, people who travel a lot, as is the case of *Mukheristas*, more than 80% of the participants in the study did not answer this question. Surprisingly, self-perception toward HPV and syphilis was low in women *Mukheristas* with a high income from *Mukhero*. They are not perceived to be at risk and vulnerability of contracting STIs/HIV, especially HPV, when they point greater risk and vulnerability to other mobility women, for example, sex workers. They also see the HIV and HPV infection as distant, confirming the idea of “other’s illness” “impossible or unlikely to happen to them”. They deny the risk, believing that HIV/AIDS and HPV threaten others and they exclude themselves from danger. A low level of vulnerability perception was observed in other contexts [31,35]. Women who self-identified themselves as high risk and vulnerability of contracting STIs/HIV were more likely to use condoms than those who were perceived as at low risk for contracting STIs/HIV [38].

Our study identified some sexual risk behaviors among women informal cross border traders, including multiple sexual partners and not using a condom. All participants with a low level of education, and low income were more likely to have had sex with multiple partners than other counterparts, most in exchange for money, goods, or services (facilitation of border crossings, cargo loading), without using a condom. These findings are consistent with another study carried out in another African country [11], and one study carried out in Mozambique [26]. 

Several reasons, which were not explored in this study, for poor adherence and inconsistent condom use have been mentioned in the literature: Enjoying sexual pleasure; familiarization of partner trust/having the habit with the client; coercive sex and rape; willingness to have children and religious beliefs; fear that the condom will break during sexual intercourse; and fear of itching and burns [39,40,41,42]. The national results of INSIDA (2009) [24] showed a low prevalence of condom use in Mozambique. Only 8% of women and 16% of men aged 15–49 who had sex in the last 12 months prior to the survey used condoms in the last sexual intercourse. Moreover, the recent national results of IMASIDA (2015) show that only 3% of women aged 15–49 years had two or more sexual partners in the last 12 months and 72% did not use condoms [25]. The lack of decision-making power that is noted in these women in relation to condom use, manifested by the transfer of responsibilities to partners, in a context of multiple and concomitant sexual partners and experiences of violence and sexual harassment during the high mobility process, has been the great challenge that these women face [43]. Therefore, condom use in the context of high mobility, where risk behaviors and vulnerabilities are increased, is a fundamental factor in the simultaneous prevention of STIs and unwanted pregnancies. 

Few participants reported signs and symptoms of STIs 30 days before the study. On the one hand, it may be that participants are not able to recognize those symptoms. On the other hand, many STIs happen without signs and symptoms that force them to stop their business. In addition, addressing sexuality and sexually transmitted diseases in the African context, particularly Mozambican, is extremely complex, namely due to the myths and cultural practices related to the sexuality [44]. For example, our participants showed many reservations to answer the questions related to gonorrhea. 

The results of this study have a useful dimension. The evidence now produced can serve as a baseline for the design of STD education campaigns adapted to this population of high cross-border mobility, which seem to be urgent. Furthermore, health promotion measures designed according to the needs of these populations will be useful to prevent infections.

This study must acknowledge some limitations. Firstly, these findings cannot be generalized, because the cross-sectional nature of our data can only demonstrate relationships between the variables. Secondly, we worked with the *Hard-to-survey population*. Thirdly, the sensibility of this topic among the WICBT in the African context, some sexual behavior may be under-reported, because we used self-reported data. Fourthly, the relatively law age and mixed gender of the enquirers could be influenced. Despite these limitations, our study provided insight about this topic that is yet unexplored in our country. 

## 5. Conclusions

This study revealed that women involved in an informal cross-border trading in the context of high mobility and circular migration in Southern Mozambique, present relatively high levels of formal education and high incomes, despite referring to the lack of schooling, poverty, and unemployment as motivation for the practice of *Mukhero*. These characteristics seem to have contributed to their social rise and could result in high levels of knowledge about STIs/HIV that would assist them in the adoption of safe sexual behavior and perception of risk and vulnerability to sexually transmitted infections, during the process of high mobility. However, low and inconsistent knowledge and misconceptions of STIs/HIV, high sexual risky behavior, low perception of risk of getting STIs/HIV, and low reported STIs symptoms, were demonstrated mostly among participants with a low education level and low income from *Mukhero*, suggesting increased risk and vulnerability to the STIs/HIV. Health promotion programs and strategies for empowerment with the knowledge for the behavioral change, contextualized to the socioeconomic and epidemiological profiles of the country, are needed to promote prevention and reduce STIs/HIV transmission targeted to this neglected and key population.

## Figures and Tables

**Table 1:** Sociodemographic characteristics, motivation for the practice of *Mukhero*, and trips’ frequency.

Variable	*n* (%)	Variable	*n* (%)
Age group		Income from *Mukhero*	
18–25	12 (6.0)	Eq. 3 minimum salaries	123 (61.5)
26–34	62 (31.0)	Eq. 4 minimum salaries	48 (24.0)
35–49	126 (63.0)	Eq. 6 minimum salaries	29 (14.5)
Total	200 (100)	Total	200 (100)
Marital status		Motivations for practicing *Mukhero*	
Single	61 (30.5)	Low school level	27 (13.5)
Married	40 (20.0)	Unemployment	49 (24.5)
Marital union	76 (39.5)	Increase family revenue	46 (23.0)
Divorced	7 (3.5)	Partner’s low salary	29 (14.5)
Widow	13 (6.5)	Divorce and widow	11 (5.5)
Missing	3 (1.5)	High fiscal taxes	3 (1.5)
		Influence of friends Influence of relatives	18 (9.0) 17 (8.5)
Total	200 (100)		200 (100)
Level of education		Start of the practice of *Mukhero*	
None	0 (0.0)	<5 years	83 (41.5)
Grade 1–5	36 (18.0)	5–10 years	55 (27.5)
Grade 6–10	114 (57)	10–15 years	27 (13.5)
Grade 11–12	33 (16.5)	15–20 years	26 (13.0)
Tertiary education	17 (8.5)	>20 years	9 (4.5)
Total	200 (100)	Total	200 (100)
Household size		Number of monthly trips	
1–3	17 (8.5)	1–3 trips	40 (20.0)
4–6	108 (54.0)	4–6 trips	135 (67.5)
≥7	63 (37.5)	>7 trips	25 (12.5)
Total	200 (100	Total	200 (100)
Main occupation			
*Mukhero*	160 (80.0)		
Other	40 (20)		
Total	200 (100)		

Abbreviations: Dk/Da: Do not know/do not answer.

**Table 2:** Respondents’ knowledge about the sexually transmitted infections (STIs).

Have Heard about the Following STIs	HIV *n* (%)	Syphilis *n* (%)	HPV *n* (%)	Gonorrhea *n* (%)	Trichomoniasis *n* (%)	Candidiasis *n* (%)
Yes	200 (100)	82 (41.0)	109 (54.5)	174 (87.4)	30 (15.0)	38 (19.0)
No	-	112 (56.0)	84 (42.0)	25 (12.5)	165 (58.0)	153 (76.5)
Dk/Da	-	6 (3.0)	7 (3.5)	1 (0.5)	5 (2.5)	9 (4.5)
Total	200 (100)	200 (100)	200 (100)	200 (100)	200 (100)	200 (100)

Abbreviations: Dk/Da: Do not know/do not answer; HIV: Human Immunodeficiency Virus; HPV: Human Papilloma Virus.

**Table 3:** Respondents’ knowledge of transmission, prevention, and testing of sexually transmitted infections (STIs).

Variable	HIV *n* (%)	Syphilis *n* (%)	HPV *n* (%)
Ways of transmission of STIs			
Sexual intercourse			
Yes	188 (94.0)	34 (17.0)	11 (5.5)
No	7 (3.5)	4 (2.0)	2 (1.0)
Dk/Da	5 (2.5)	162 (81.0)	187 (93.5)
Total	200 (100)	200 (100)	200 (100)
Sexual intercourse without protection			
Yes	192 (96.0)	34 (17.0)	11 (5.5)
No	4 (2.0)	3 (1.5)	2 (1.0)
Dk/Da	4 (2.0)	163 (81.5)	187 (93.5)
Total	200 (100)	200 (100)	200 (100)
Sexual intercourse without protection with various partners			
Yes	175 (87.5)	29 (14.5)	10 (5.0)
No	18 (9.0)	6 (3.0)	2 (1.0)
Dk/Da	7 (3.5)	165 (82.5)	188 (94.0)
Total	200 (100)	200 (100)	200 (100)
Through mosquito bite			
Yes	44 (22.0)	1 (0.5)	1 (0.5)
No	126 (63.0)	11 (5.5)	11 (5.5)
Dk/Da	30 (15.0)	188 (94.0)	188 (94.0)
Total	200 (100)	200 (100)	200 (100)
Ways of prevention of STIs	HIV	Syphilis	HPV
Regular condom use			
Yes	195 (97.5)	36 (18.0)	17 (8.5)
No	2 (1.0)	-	4 (2.0)
Dk/Da	3 (1.5)	164	179 (89.5)
Total	200 (100)	200 (100)	200 (100)
Reduction of the number of sexual partners			
Yes	177 (88.5)	36 (18.0)	16 (8.0)
No	18 (9.0)	-	5 (2.5)
Dk/Da	5 (2.5)	164	179 (89.5)
Total	200 (100)	200 (100)	200 (100)
To have only one sexual partner not infected			
Yes	181 (90.5)	36 (18.0)	16 (8.0)
No	14 (7.0)	-	5 (2.5)
Dk/Da	5 (2.5)	164	179 (89.5)
Total	200 (100)	200 (100)	200 (100)
To have sexual intercourse with several partners			
Yes	29 (14.4)	5 (2.5)	1 (0.5)
No	166 (70.0)	29 (14.5)	13 (6.5)
Dk/Da	5 (2.5)	166 (83.0)	186 (93.9)
Total	200(100)	200 (100)	200 (100)
To have sexual intercourse with virgin girls and boys			
Yes	39 (19.5)	5 (2.5)	1 (0.5)
No	140 (70.0)	29 (14.5)	13 (6.5)
Dk/Da	21 (10.5)	166 (83.0)	186 (93.9)
Total	200 (100)	200 (100)	200 (100)
Testing for the following STIs			
Yes	85.0	10.0	(10.0)
No	15.0	78.5	(78.5)
Dk/Da	-	11.5	(11.5)
Total	200 (100)	200 (100)	200 (100)

Abbreviations: Dk/Da: Do not know/do not answer; HIV: Human Immunodeficiency Virus; HPV: Human Papilloma Virus.

**Table 4:** Respondents’ perception about the possibility of contracting sexually transmitted infections (STIs).

Possibility	HIV *n* (%)	Syphilis *n* (%)	HPV *n* (%)
High possibility	133 (66.5)	8 (4.0)	10 (8.1)
Low possibility	55 (27.5)	27 (13.5)	7 (5.6)
Dk/Da	12 (6.0)	165 (82.5)	183 (86.3)
Total	200 (100)	200 (100)	200 (100)

Abbreviations: Dk/Da: Do not know/do not answer; HIV: Human Immunodeficiency Virus; HPV: Human Papilloma Virus.

**Table 5:** Respondents’ sexual risk behaviors and, reported signs and symptoms of sexually transmitted infections (STIs).

Variable	*n*	%
How many sexual partners have you had in the last 12 months?		
0	2	1.0
1	156	78.0
>2	22	11.0
Dk/Da	20	10.0
Total	200	100
Did you have occasional sexual intercourse in the past 12 months?		
Yes	30	15.0
No	168	84.0
Dk/Da	2	1.0
Total	200	100
Did you always use a condom?		
Yes	16	53.3
No	9	30.0
Dk/Da	5	16.7
Total	30	100
Have you ever had sex in exchange for money/goods/services?		
Yes	15	7.5
No	184	92.0
Dk/Da	1	0.5
Total	200	100
Sex in exchange for services		
Yes	14	99.5
No	1	0.5
Dk/Da	-	-
Total	15	100
Reported signs and symptoms of STIs		
*Vaginal discharge*		
Yes	34	17.0
No	165	82.5
Dk/Da	1	0.5
Total	200	100
*Pain in the lower abdomen*		
Yes	38	19.0
No	161	80.5
Dk/Da	1	0.5
Total	200	100
*Genital itching (itching)*		
Yes	18	9.0
No	179	89.0
Dk/Da	3	1.5
Total	200	100
*Ulcer/wound/genital tumor*		
Yes	6	3.0
No	192	96.0
Dk/Da	2	1.0
Total	200	100

Abbreviations: Dk/Da: Do not know/do not answer.

**Table 6:** Bivariate analysis between knowledge, risk perception, sexual behaviors about STIs/HIV and education level, marital status, and income from *Mukhero*.

Variable	*n* (%)	Education Level			*n* (%)	Marital Status			*n* (%)	Income from *Mukhero*		
		High	Low	*p*		Married	Not Married	*p*		High	Low	*p*
Knowledge about STIs/HIV												
Has heard about Gonorrhea	*n* = 199	*n* = 50	*n* = 149									
Yes	174 (87.4)	48 (96.0)	126 (84.6)	<0.001		-	-	-				
No	25 (12.6)	2 (4.0)	23 (15.4)			-	-					
Has heard about HPV									*n* = 193	*n* = 28	*n* = 165	
Yes	-	-	-	-		-	-	-	109 (56.5)	16 (57.1)	93 (56.4)	<0.001
No	-	-	-				-		84 (43.5)	12 (42.9)	72 (43.6)	
Has heard about Syphilis	*n* = 194	*n* = 50	*n* = 144									
Yes	82 (42.3)	33 (66.0)	49 (34.0)	<0.001		-	-	-				
No	112 (57.7)	17 (34.0)	95 (66.0)									
Has heard about Candidiasis	*n* = 191	*n* = 49	*n* = 142		*n* = 191	*n* = 113	*n* = 78					
Yes	38 (19.9)	17 (34.7)	21 (14.79)	<0.001	38 (19.9)	19 (16.8)	19 (24.4)	0.021				
No	153 (80.1)	32 (65,3)	121 (85.21)		153 (80.1)	94 (83.2))	59 (75.6)					
Has heard about Tricomoniasis					*n* = 195	*n* = 117	*n* = 78					
Yes	-	-	-	-	30 (15.4)	13 (11.1)	17 (21.8)	0.028				
No	-	-	-		165 (84.6)	104 (88.9)	61 (78.2)					
Sex with one uninfected partner prevents HIV					*n* = 195	*n* = 116	*n* = 79					
Yes	-	-	-	-	181 (92.8)	104 (86.6)	77 (97.5)	0.047				
No	-	-			14 (7.2)	12 (10.4)	2 (2.5)					
Mosquito’s bite spreads HIV	*n* = 171	*n* = 45	*n* = 126									
Yes	44 (25.73)	4 (8,9)	40 (31.75)	<0.001								
No	127 (74.27)	41 (91.0)	86 (68.25)									
Drink/share some cup or toilet spreads HIV	-	-	-	-	*n* = 193	*n* = 116	*n* = 77					
Yes	-	-	-	-	46 (23.8)	20 (17.2)	26 (33.8)	0.009		-	-	-
No	-	-	-	-	147 (76.2)	96 (82.8)	51 (66.2)			-	-	
Unprotected sex with multiple partners spreads Syphilis	*n* = 35	*n* = 12	*n* = 23									
Yes	29 (82.9)	11 (91.7)	18 (78.3)	0.006		-	-	-				
No	6 (17.1)	1 (8.3)	5 (21.7)			-	-					
Sex with one uninfected partner prevents Syphilis									*n* = 195	*n* = 29	*n* = 166	
Yes	-	-	-	-	-	-	-	-	181 (92.8)	23 (79.3)	158 (95.2)	0.027
No	-	-	-	-	-	-	-	-	14 (7.2)	6 (20.7)	8 (4.8)	
Sex with virgin young boys prevents HIV	*n* = 170	*n* = 46	*n* = 124		*n* = 170	*n* = 98	*n* = 72	0.038				
Yes	32 (18.8)	1 (2.2)	31 (25.0)	0.006	32 (18.8)	26 (26.5)	6 (8.3)					
No	138 (81.2)	45 (97.8)	93 (75.0)		138 (81.2)	72 (73.5)	66 (91.7)					
Sex with virgin young girl prevents HIV	*n* = 170	*n* = 46	*n* = 124	0.007	-	-	-	-				
	*n* = 179	*n* = 46	*n* = 133		-	-	-					
Yes	39 (21.8)	1 (2.2)	38 (28,6)	0.007	-	-	-					
No	140 (78.2)	45 (97.8)	95 (71.4)		-	-						
*Perception of getting STIs/HIV*												
Perception of getting HIV/AIDS by *Mukheristas*	*n* = 197	*n* = 50	*n* = 147									
High risk	133 (67.5)	43 (86.0)	90 (61.2)		-	-	-					
Low risk	55 (27.9))	4 (8.0)	51 (34.7)	0.015	-	-	-					
Dk/Da	9 (4.6)	3 (6.0)	6 (4.1)		-	-	-					
Perception of getting HPV by sex workers									*n* = 186	*n* = 26	*n* = 160	
High risk	-	-	-	-	-	-	-		176 (94.6)	26 (100.0)	150 (93.8)	0.036
Low risk		-	-			-	-	-	10 (5.4)	0 (0.0)	10 (6.2)	
Perception of getting Syphilis by *Mukheristas*	*n* = 200	*n* = 50	*n* = 150						*n* = 200	*n* = 29	*n* = 171	
High risk	27 (13.5)	12 (24.0)	15 (10.0)	<0.001		-	-	-	27 (13.5)	9 (31.1)	18 (10.5)	0.022
Low risk	8 (4.0)	1 (2.0)	7 (4.7)			-	-		8 (4.0)	1 (3.4)	7 (4.1)	
Dk/Da	165 (82.5)	37 (74.0)	128 (85.3)			-	-		165 (82.5)	19 (65.5)	146 (85.4)	
Perception of getting HPV by *Mukheristas*	*n* = 124	*n* = 35	*n* = 89						*n* = 27	*n* = 158	*n* = 185	
High risk	7 (5.6)	6 (17.1)	1 (1.1)			-	-	-	20 (74.1)	121 (76.6)	141 (76.2)	0.041
Low risk	10 (8.1)	1 (2.9)	9 (10.1)	0.01		-	-		7 (25.9)	37 (23.4)	44 (23.8)	
Dk/Da	107 (86.3)	28 (80.0)	79 (88.8)			-	-					
*Testing STIs/HIV*												
Ever tested for HIV	*n* = 199	*n* = 50	*n* = 149						*n* = 199	*n* = 29	*n* = 170	
Yes	170 (85.4)	47 (94.0)	123 (82.6)	0.046		-	-	-	170 (85.4)	27 (93.1)	143 (84.1)	0.011
No	29 (14.6)	3 (6.0)	26 (17.4)			-	-		29 (14.5)	2 (6.9)	27 (15.9)	
Ever tested for Syphilis	*n* = 177	*n* = 44	*n* = 133									
Yes	20 (11.3)	10 (22.7)	10 (7.5)	0.003		-	-	-				
No	157 (88.7)	34 (77.3)	123 (92.5)			-	-					
*Risk sexual behaviors*												
Ever had sex in exchange for money/goods/services	*n* = 15	*n* = 6	*n* = 9						*n* = 199	*n* = 28	*n* = 171	
Yes	1 (6.7)	0 (0.0)	1 (11.1)	0.01		-	-	-	15 (7.5)	0 (0.0)	15 (8.8)	0.042
No	14 (93.3)	6 (100.0)	8 (88.9)			-	-		184 (92.5)	28 (100,0)	156 (91.2)	

HIV: Human Immunodeficiency Virus; HPV: Human Papilloma Virus; STIs: Sexually transmitted infections.
